# Efficiency of Sorting Site of Care for Frail Patients Undergoing Mastectomy

**DOI:** 10.1245/s10434-025-18910-5

**Published:** 2026-01-14

**Authors:** Claire R. Morton, Yu-Jen Chen, Kenneth Williams, Randall A. Bloch, Ezra S. Brooks, Christina Minami, Louis L. Nguyen

**Affiliations:** 1 Center for Surgery and Public Health, Brigham and Women’s Hospital, Boston, MA USA; 2 Patient-Reported Outcomes, Value, and Experience (PROVE) Center, Brigham and Women’s Hospital, Boston, MA USA; 3 Department of Surgery, Brigham and Women’s Hospital, Boston, MA USA; 4 Breast Oncology Program, Dana-Farber/Brigham and Women’s Hospital, Boston, MA USA

**Keywords:** Frailty, Mastectomy, Ambulatory surgery, Site of care

## Abstract

**Background:**

Patients undergo mastectomy in both ambulatory surgery centers (ASCs) and inpatient settings. Guidelines for site selection are poorly defined. Older adults, particularly those with frailty, are at increased risk of adverse outcomes postoperatively. Transfer to an acute hospital is a unique adverse event suggesting potentially inappropriate ASC care.

**Methods:**

The authors used logistic regression modeling to describe the association of frailty with site of care and transfer, and modeled expected costs associated with ambulatory mastectomy for robust and prefrail or frail patients.

**Results:**

In ASCs, 85.3% of all patients and 51.3% of prefrail or frail patients underwent mastectomy. Frailty or prefrailty was associated with increased odds of inpatient care (odds ratio [OR], 5.856; *p* < 0.001). Odds of transfer were higher among prefrail and frail patients (OR, 2.640; *p* < 0.05), but rates remained low (< 0.4%). Rates of transfer needed to negate cost-savings from ambulatory procedures are more than 100 times the current rate (38%; standard error, 4.7%). If all prefrail and frail patients received care at ASCs, expected cost savings would be $8404 per patient.

**Conclusions:**

Despite slightly higher rates of transfer, clinicians should consider treating frail and prefrail older adults in ASCs given possible economic benefits.

Breast cancer is diagnosed for approximately 300,000 patients annually in the United States, approximately one third of whom undergo mastectomy.^1,2^ Patients undergo mastectomies in a number of sites, including freestanding ambulatory surgery centers (ASCs) and hospital outpatient departments (HOPDs). For appropriate patients,^3–5^ ASCs are considered safe, and care in ASCs is associated with significant cost savings relative to care at hospitals,^6^ contributing to ongoing shifts in site-of-care selection.

Financially constrained health care systems, patient preference, and overcrowded hospital wards all have contributed to an increasing proportion of mastectomies being performed on an ambulatory basis.^6^ Despite this shift toward ASC care, there is no accepted metric for determining a patient’s approriateness for ambulatory surgery.

Frailty, a clinical syndrome marked by decreased physiologic reserve and increased vulnerability to stressors, is present in an estimated 43% of women with breast cancer.^7–9^

Frail patients are more likely to experience complications and to use a greater amount of health care resources after surgery than robust patients.^10–13^ As a result, frailty assessments are becoming increasingly integrated into surgical practice. The interplay of frailty and appropriateness for different sites of care, however, remains under-explored.

In previous studies, clinicians appeared to be triaging patients with greater multimorbidity to settings with greater postoperative supervision.^14^ However, the impact of frailty, a distinct entity influenced by multimorbidity but not determined by it, on site of care has not been explicitly described to date. Furthermore, although ASCs are widely considered safe, frailty has not been investigated as a risk factor for undertriage to ASC care, including transfer to acute-care hospitals after surgery, a key quality metric. Finally, although studies reported in prior literature have investigated costs associated with ambulatory and inpatient care,^6^ analyses that take into account differential risk profiles for frail patients or that establish recommended care patterns have yet to be conducted.

Our investigation had three aims: (1) to characterize the current impact of frailty on site-of-care selection, (2) to describe the potential effects of undertriage to ASC care based on frailty status, and (3) to clarify economically optimal care patterns for patients undergoing mastectomy. Our results may ultimately be used to guide practice recommendations and inform further policy changes regarding coverage of inpatient stays associated with mastectomy.

## Methods

### Data Source

We used the Agency for Healthcare Research and Quality (AHRQ) Healthcare Cost and Utilization Program’s Nationwide Inpatient Sample (NIS) and Nationwide Ambulatory Surgery Sample (NASS) databases from 2019 to 2022. The project was deemed non-human subjects research by the Massachusetts General Brigham Institutional Review Board and exempted from further review.

### Study Population

Both databases (NIS and NASS) were queried for all female patients age 18 years or older undergoing mastectomy for a diagnosis of breast cancer. Although frailty is more common in those older than 65 years, we included all adult women to capture any patients who may meet criteria for frailty despite being younger than 65 years. We excluded patients with metastatic cancer and those receiving concurrent reconstruction because these patients may have alternative factors playing a role in the clinician’s decision to treat them as inpatients. We identified ambulatory operations as recorded in the NASS and inpatient surgeries as those recorded in the NIS. Procedure codes used were those used in prior studies to identify similar samples of patients.^15,16^

### Variables

We collected demographic variables, including patient age, race, and urbanicity. We also considered clinical variables, including hospital teaching status. We defined frailty using the Hospital Frailty Risk Score,^17^ which stratifies patients on the basis of the International Classification of Diseases, 10th revision (ICD-10)-coded comorbidities into low-risk patients with a score lower than 5 (robust), intermediate-risk patients with a score of 5 to 15 (prefrail), or high-risk patients with a score higher than 15 (frail). We grouped prefrail and frail patients given limited numbers of patients in these groups and prior studies suggesting minimal difference in outcomes between medium and high risk of frailty groups.^18,19^

### Outcomes

The primary outcome was location of surgery determined by data file source, with inpatient surgery identified in the NIS and ASC surgeries identified via the NASS. The NASS captures ambulatory surgeries performed in hospital-owned facilities independent of location and may include observation stays. The secondary outcome, transfer to an acute-care hospital, was assessed only among patients receiving care in an ambulatory setting. Transfer to an acute-care hospital after ambulatory procedures was selected because it is a rare but essential quality indictor suggesting unsuitability of ambulatory care.^20–22^ Costs of care by site were calculated via weighted means from sample data per protocol surggested by the AHRQ.

### Analysis

#### Impact of Frailty on Site-of-Care Selection and Transfer After Ambulatory Surgery

Tables 1 and Table 3 summarize categorical variables using frequencies with percentages and continuous variables using medians and interquartile ranges (IQRs). We conducted descriptive analyses using chi-square tests to compare categorical characteristics and Wilcoxon rank-sum tests for continuous variables by site of care and post-surgery transfer to an acute-care hospital, respectively. Subsequently, we performed adjusted multivariable logistic regression to assess the associations of frailty with site of care, accounting for demographic (age, race, urbanicity) and clinical (location and teaching status) variables.

**Table 1:** Sample and demographic information

	Total (*n* = 222,943) *n* (%)	Inpatient (*n* = 32,795, 14.7%) *n* (%)	Ambulatory (*n* = 190,148, (85.3%) *n* (%)	*p* Value
Median age; years (IQR)	65.1 (54.8–73.3)	64.9 (54.4–73.8)	65.1 (54.9–73.2)	0.0495
Race (*n*), row (%)				< 0.0001
White	148,466 (66.6)	19,200 (12.9)	129,266 (87.1)	
Black	27,713 (12.4)	5370 (19.4)	22,343 (80.6)	
Hispanic	21,326 (9.6)	4035 (18.9)	17,291 (81.1)	
Others	25,438 (11.4)	4190 (16.5)	21,248 (83.5)	
Urban/rural				< 0.0001
Metropolitan	183,295 (82.2)	28,290 (15.4)	155,005 (84.6)	
Micropolitan	22,455 (10.1)	2560 (11.4)	19,895 (88.6)	
Not metropolitan or micropolitan	17,193 (7.7)	1945 (11.3)	15,248 (88.7)	
Location/teaching status of hospital				0.1373
Urban teaching	165,250 (74.1)	24,665 (14.9)	140,585 (85.1)	
Urban non-teaching	38,459 (17.3)	5525 (14.4)	32,934 (85.6)	
Rural	19,233 (8.6)	2605 (13.5)	16,628 (86.5)	
Median Hospital Frailty Risk Score (IQR)	0 (0.0–1.5)	0.7 (0.0–2.1)	0 (0.0–1.4)	< 0.0001
Hospital frailty risk score				< 0.0001
< 5 (robust)	217,890 (97.7)	30,345 (13.9)	187,545 (86.1)	
≥ 5 (prefrail & frail)	5053 (2.3)	2450 (48.5)	2603 (51.5)	
Year				< 0.0001
2019	56,804 (25.5)	10,745 (18.9)	46,059 (81.1)	
2020	52,772 (23.7)	7835 (14.8)	44,937 (85.2)	
2021	55,318 (24.8)	7185 (13.0)	48,133 (87.0)	
2022	58,049 (26.0)	7030 (12.1)	51,019 (87.9)	

IQR, Interquartile range

Additional subset analysis were performed separately in the robust population and the prefrail and frail population to identify factors influencing site-of-care selection within each group. We further examined factors associated with transfer to an acute-care facility after care in an ASC using an adjusted logistic regression model, accounting for the same demographic and clinical covariates. All analyses accounted for the complex survey design, including stratification and clustering. We applied discharge weights to produce national estimates. The analyses were performed with SAS software, v.9.4 (SAS Institute, Cary, NC, USA).

#### Econometric Analysis

For our econometric analysis, we used a decision tree to characterize clinician site-of-care selection and to determine expected costs associated with each site of care for women with breast cancer requiring a mastectomy. Expected costs were calculated based on weighted average charges by location, documented proportions of patients receiving care in both locations by frailty status, and predicted probabilities of transfer from the previously described logistic regression models using the following formula:

1 $$\begin{aligned} {\mathrm{Total}}\;{\mathrm{expected}}\;{\mathrm{cost}} & = {\mathrm{cost}}^{{{\mathrm{ASC}}}} \times {\mathrm{proportion}}^{{{\mathrm{ASC}}}} + {\mathrm{cost}}^{{{\mathrm{inpatient}}}} \times \left( {{1} - {\mathrm{proportion}}^{{{\mathrm{ASC}}}} } \right) \\ & \quad + \;{\mathrm{cost}}^{{{\mathrm{transfer}}}} \times {\mathrm{probability}}^{{{\mathrm{transfer}}}} . \\ \end{aligned}$$

Charges rather than patient-facing costs were used to refect the economic efficiency of the system rather than the burden to the patient. Cost of transfer was estimated to be $45,390 (standard error [SE], $5,518.93) based on prior literature reporting the cost differential between ambulatory surgery requiring a readmission after the procedure and ambulatory surgery that did not require a readmission after the procedure.^6^ The model relies on the simplifying assumption that care is provided in either inpatient settings or ambulatory surgical settings, aligned with how we analyzed and interpreted our data. Because care at an ASC costs less than inpatient care, cost savings were estimated based on assuming constant rates of transfer while transitioning all mastectomy care to ASCs (i.e., setting proportion^ASC^ to 1. Maximum allowable transfer risk was determined to be the rate of transfer such that expected cost savings for total ASC care were equivalent to expected costs associated with transfer to inpatient care. These analyses were performed in R version 4.4.1(Vienna, Austria).

## Results

### Overview

The sample consisted of 222,943 patients, a minority of whom (14.7%) underwent their mastectomy in inpatient settings (Table 1). The median age was 65.1 years (IQR, 54.8–73.3 years). Most patients (66.6%) were white and lived in metropolitan settings (82.2%). Most treating facilities were urban teaching centers (74.1%). Most patients (97.7%) were robust.

#### Impact of Frailty on Site-of-Care Selection and Transfer After Ambulatory Surgery

Patients receiving inpatient care were younger (median age, 64.9 years; IQR, 54.4–73.8 years) than patients receiving care at ASCs (median age, 65.1 years; IQR, 54.9–73.2 years; *p* = 0.05). The proportion of patients receiving inpatient care was highest among black patients (19.4%) and lowest among white patients (12.9%) (*p* < 0.001). Patients in metropolitan areas were more likely to receive care in inpatient settings (15.4%) than those in micropolitan settings (11.4%; *p* < 0.001). There were no significant differences in the proportion of patients receiving care in either setting based on location or teaching status of the hospital. Patients who were frail or prefrail were more likely to receive care in inpatient settings than robust patients (48.5% vs 13.9%; *p* < 0.001), and median frailty scores were significantly higher in the inpatient population (0.7 [IQR, 0.0–2.1] vs 0 [IQR, 0.0–1.4]; *p* < 0.001).

In multivariable analysis, prefrailty and frailty were associated with a 5.86 increase in odds (95% CI 5.21–6.59; *p* < 0.001) of inpatient surgery relative to robust patients when adjustment for clinical and demographic variables was used (Table 2). Patients who were black (OR, 1.54; 95% CI 1.40–1.70), Hispanic (OR, 1.56; 95% CI 1.38–1.76), or other (OR, 1.32; 95% CI 1.17–1.48) were more likely to receive inpatient care than white patients (*p* < 0.001). Patients receiving care in non-metropolitan areas were less likely to receive inpatient care (micropolitan: OR, 0.65; 95% CI 0.57–0.74; not metropolitan or micropolitan: OR, 0.68; 95% CI 0.59–0.78; *p* < 0.001). Patients receiving care at hospitals in rural areas were more likely to receive inpatient care (OR, 1.33; 95% CI 1.15–1.53; *p* < 0.001). Among robust patients, factors associated with increased odds of inpatient care included non-white race, urbanicity, and rural hospital location (*p* < 0.001). Among prefrail and frail patients, only black patients were more likely to receive inpatient care (OR, 1.44; 95% CI 1.12–1.86; *p* < 0.001).

**Table 2:** Multivariable analysis of factors associated with receiving inpatient care

Total sample (*n* = 222,943)		
	OR (95% CI)	*p* Value
Hospital frailty risk score		
Robust	Reference	
Prefrail and frail	5.856 (5.205–6.588)	**< 0.0001**
Age	1.001 (0.998–1.003)	0.6356
Race		
White	Reference	
Black	1.541 (1.399–1.697)	**< 0.0001**
Hispanic	1.555 (1.376–1.758)	**< 0.0001**
Others	1.316 (1.174–1.476)	**< 0.0001**
Urban/rural		
Metropolitan	Reference	
Micropolitan	0.645 (0.565–0.736)	**< 0.0001**
Not metropolitan or micropolitan	0.675 (0.585–0.778)	**< 0.0001**
Location/teaching status of hospital		
Urban teaching	Reference	
Urban non-teaching	0.962 (0.871–1.064)	0.4517
Rural	1.328 (1.153–1.530)	**< 0.0001**
Robust subset (*n* = 217,890)		
Age	1.001 (0.998–1.003)	0.4823
Race		
White	Reference	
Black	1.546 (1.400–1.708)	**< 0.0001**
Hispanic	1.568 (1.386–1.775)	**< 0.0001**
Others	1.323 (1.178–1.486)	**< 0.0001**
Urban/rural		
Metropolitan	Reference	
Micropolitan	0.619 (0.540–0.710)	**< 0.0001**
Not metropolitan or micropolitan	0.662 (0.572–0.767)	**< 0.0001**
Location/teaching status of hospital		
Urban teaching	Reference	
Urban non-teaching	0.957 (0.866–1.059)	0.3966
Rural	1.367 (1.184–1.577)	**< 0.0001**
Prefrail and frail subset (*n* = 5053)		
Age	0.993 (0.985–1.002)	0.1218
Race		
White	Reference	
Black	1.440 (1.115–1.860)	**0.0053**
Hispanic	1.205 (0.819–1.772)	0.3431
Others	1.132 (0.790–1.623)	0.4981
Urban/rural		
Metropolitan	Reference	
Micropolitan	1.380 (0.918–2.073)	0.1214
Not metropolitan or micropolitan	0.971 (0.640–1.474)	0.8911
Location/teaching status of hospital		
Urban teaching	Reference	
Urban non-teaching	0.756 (0.530–1.079)	0.4035
Rural	1.064 (0.920–1.230)	0.1230

OR, Odds ratio; CI, Confidence interval

Bold values are statistically significant with a *p* value < 0.05

Of patients treated in an ASC (190,148), 209 were transferred to an acute hospital after their procedure (0.1%) (Table 3). Age, race, urbanicity, location, and teaching status of the hospital and frailty all were associated with transfer after surgical treatment (Table 3). In multivariable analysis, being prefrail or frail (OR, 2.64; 95% CI 1.14–6.13; *p* < 0.05) and receiving care at a rural hospital (OR, 5.41; 95% CI 2.73–10.78; *p* < 0.001) were associated with increased odds of transfer, with adjustment for age, race, and urbanicity (Table 4).

**Table 3:** Association of transferring to acute-care hospitals and frailty among ASC patients

Ambulatory (*n* = 190,148)	Transferring to acute-care hospitals		*p* Value
	Yes (*n* = 209, 0.1%) *n* (%)	No (*n* = 189,938, 99.9%) *n* (%)	
Median age: years (IQR)	68.1, 59.4–76.2	65.1, 54.9–73.2	0.0020
Race (*n*), row (%)			0.0403
White	166 (79.2)	129,100 (68.0)	
Black	19 (9.2)	22,324 (11.7)	
Others	24 (11.6)	38,514 (20.3)	
Urban/rural			< 0.0001
Metropolitan	121 (57.6)	154,884 (81.5)	
Micropolitan	44 (21.1)	19,850 (10.5)	
Not metropolitan or micropolitan	45 (21.3)	15,203 (8.0)	
Location/teaching status of hospital			< 0.0001
Urban teaching	96 (45.7)	140,489 (74.0)	
Urban non-teaching	38 (18.4)	32,895 (17.3)	
Rural	75 (35.9)	16,553 (8.7)	
Median hospital frailty risk score (IQR)	0, 0–1.4	0, 0–1.4	0.0005
Hospital frailty risk score			0.0021
< 5 (robust)	≥ 200 (≥ 95%)	187,344 (98.6)	
≥ 5 (prefrail and frail)	≤ 11 (≤ 5%)^a^	2594 (1.4)	
Year			0.6153
2019	59 (28.0)	46,000 (24.2)	
2020	56 (26.9)	44,881 (23.6)	
2021	46 (22.1)	48,087 (25.3)	
2022	48 (22.9)	50,971 (26.8)	

ASC, Ambulatory surgery center; IQR, Interquartile range

^a^Per Healthcare Cost and Utilization Project (HCUP) data use agreement; cell sizes < 10 are suppressed

**Table 4:** Multivariable analysis of factors associated with transferring to acute-care hospitals among ASC patients

*n* = 190,148	OR (95% CI)	*p* Value
Hospital frailty risk score		
Robust	Reference	
Prefrail and frail	2.640 (1.138–6.127)	**0.0238**
Age	1.012 (0.998–1.027)	0.0974
Race		
White	Reference	
Black	0.890 (0.486–1.630)	0.7055
Others	0.678 (0.344–1.339)	0.2632
Urban/rural		
Metropolitan	Reference	
Micropolitan	0.951 (0.487–1.856)	0.8829
Not metropolitan or micropolitan	1.515 (0.792–2.898)	0.2098
Location/teaching status of hospital		
Urban teaching	Reference	
Urban non-teaching	1.650 (1.028–2.647)	**0.0379**
Rural	5.417 (2.725–10.769)	**< 0.0001**

ASC, Ambulatory surgery center; OR, Odds ratio; CI, Confidence interval

Bold values are statistically significant with a < 0.05

#### Expected Costs, Cost Savings, and Maximum Allowable Transfer Risk

Weighted average costs associated with mastectomy were $64,799.74 (SE, $808.40) for inpatient care and $47,472.71 (SE, $102.32) for ASC care. Estimated probability of transfer was 0.107% (SE, 0.001%) among robust patients and less than 0.4% (SE, 0.01%) among prefrail and frail patients. Total expected costs per patient associated with undergoing mastectomy were $49,929.54 (SE, $142.89) among robust patients and $56,036.23 (SE, $396.05) among prefrail and frail patients. If all prefrail and frail patients underwent ambulatory surgery with transfer rates at currently observed levels, total expected costs would be $47,632.62 (SE, $104.00), yielding an expected cost savings of $8404 per patient. The maximum allowable transfer risk such that there would be expected cost equivalency between ASC and inpatient care would be 38% (SE, 4.7%), more than 100 times greater than currently observed transfer rates seen among frail and prefrail patients.

## Discussion

We found that among patients undergoing mastectomy, frailty is associated with inpatient surgery as opposed to ASC surgery. Overall, although transfer to an inpatient hospital after mastectomy in an ASC was a rare event in either population, transfer was more common among prefrail and frail patients. Average costs associated with inpatient care were higher than those associated with ASC care. Expected costs for robust patients were lower than for prefrail and frail patients, attributable to the described differences in site-of-care selection. We describe opportunities for cost savings in excess of $8000 per patient if all prefrail and frail patients were to undergo mastectomy in ASCs rather than inpatient settings.

Finally, we demonstrated that for expected costs between the two sites to be equivalent, the transfer rate would need to be approximately 100 times greater than is currently observed among prefrail and frail patients.

In our study, we found that prefrail and frail patients were more likely to undergo mastectomy in inpatient settings. This is consistent with prior studies that have described clinicians selecting lower-risk patients for treatment in ASCs.^23–25^ Our study was, however, not able to disentangle the numerous factors that fuel site-of-care selection, including clinician judgment and patient preference. For example, site-of-care selection often involves a collaborative process between anesthesiologists and surgeons and may be further influenced by institutional protocols that dictate who may or may not receive care in ambulatory surgery centers. Clinicians preferentially selecting prefrail and frail patients to undergo inpatient surgery may represent a laudable attempt to provide greater support to patients. However, inpatient stays of frail older adults also are linked to delirium, which can independently confer significant long-term consequences.^26^ Prior work has already highlighted potential cost savings and psychological benefits of ambulatory surgery for breast cancer for all patients.^27^ Our results suggest that prefrail and frail patients are potentially being over-triaged to undergo inpatient care.

We also observed an association between race and inpatient surgery, with non-white patients more likely to undergo inpatient surgery. This impact was stronger among robust patients, with black, Hispanic, and patients identified as “other” all more likely to undergo inpatient surgery. Among prefrail and frail patients, however, a statistically significant impact was seen only among black patients. These findings suggest that frailty may be a greater driver for site of care than race in certain groups.

Overall rates of transfer to an acute-care hospital after ASC mastectomy were exceptionally low. Although patients with prefrailty or frailty had significantly higher odds of transfer statistically, rates of transfer remained exceptionally low (< 0.5%). These low rates of transfer to acute care at the time of ASC discharge are similar to previously reported figures.^20–22^ Low rates of transfer, even among prefrail and frail patients, are reassuring and support the expansion of ASC care within this category of older adults.

Average costs of care were higher for inpatient versus ambulatory mastectomy, aligned with the cost savings associated with ambulatory breast cancer care seen in other studies.^6,27^ Our study adds to the literature by describing differences in expected costs, taking into account transfer to acute care after ASC surgery to create a more nuanced understanding of total costs associated with ASC care. Given low rates of transfer, the cost savings associated with ASC care remained significant. Our analyses further demonstrated that taking current rates of transfer as given, health systems have significant opportunities to economically optimize the site of care, including that for prefrail and frail older adults, by expanding access to ASC care.

Our study captured only the economic costs associated with care, creating a limited picture of relative advantages or disadvantages of each site of care. Prior work has highlighted the importance of capturing patient perspectives on ASC care, a domain than remains understudied.^28^ Previous research also has highlighted potentially uncaptured benefits, including psychological well-being^27^ and uncaptured costs, such as caregiver strain,^29^ associated with ASC care. Future work with a focus on capturing the patient and care partner perspective in the perioperative period, as well as describing features of more successful facilities, may strengthen the argument for tranisitioning more care to ASC settings.

Additionally, this analysis did not include facility level detail about the services available at each ASC, which can exhibit variability in terms of proximity to inpatient hospitals, availability of overnight stays, or access to additional services (e.g., radiology), which may influence suitability of ASC care for a given patient. Our model relies on a number of simplications, such as considering care only as inpatient or ASC care, which does not capture the role of HOPDs, or observation-only inpatient stays or use of a limited number of factors driving the site-of-care selection.

Our study also was limited by the shortcomings of a claims-based frailty index, which captures comorbidity rather than clinical frailty. Our data were limited. We were not able to assess reasons for transfer, nor were we able to assess outcomes related to the post-transfer hospitalization, including length of stay. In using readmission costs as a proxy for transfer costs, we may have either over- or underestimated how much a transfer alone would have cost. Transfer costs may vary with distance and outcome of transfer, so they may not apply universally.

Additionally, our analysis did not include an investigation of the impact of primary payor and insurance coverage, which may influence where patients are eligible to receive care, nor were we able to perform state-based assessments within either national database. Finally, we assumed stable transfer risks with expanding pools of patients. but if the highest-risk patients are currently undergoing is inpatient surgery, the transfer risk may rise. In sensitivity analyses, however, this rate would need to be more than 100 times the currently observed rate to violate economic equivalence between sites of care. Shifts in care patterns during the study period between 2019 and 2022 may have favored ambulatory care, potentially limiting our estimate of potential savings if surgeons were diverting more patients to ASCs who during non-pandemic conditions may have been received inpatient treatment.

Our findings suggest that at least from an economic perspective, clinicians should consider recommending ambulatory surgery to more prefrail and frail patients. As ASC mastectomy care becomes more common and is established as safe for an increasing portion of the population, states may need to revisit policies mandating inpatient coverage after breast surgery,^30^ weighing the need to ensure that appropriate treatment remains available for patients best cared for in the hospital and that ASCs are available with appropriate capacity including pathology or nuclear medicine for axillary breast surgery. Furthermore, as care for increasingly vulnerable patients shifts to the outpatient setting, developing supports in the community will be imperative.
